# “Women Friendly”: A Childbirth Preparation Intervention in Israel for Women with Symptoms of Post-Traumatic Stress Disorder

**DOI:** 10.3390/ijerph20196851

**Published:** 2023-09-28

**Authors:** Rachel Bachner-Melman, Racheli Haim-Dahan, Ada H. Zohar

**Affiliations:** 1 Ruppin Academic Center, Emek Hefer 4025000, Israel; 2 School of Social Work, Hebrew University of Jerusalem, Jerusalem 9190500, Israel; 3 Lior Zfaty Suicide and Mental Pain Research Center, Ruppin Academic Center, Emek Hefer 4025000, Israel

**Keywords:** childbirth preparation, pregnancy, childbirth, PTSD, fear of childbirth, labor, staff training, midwives, secondary traumatic stress, “Women Friendly”

## Abstract

Pregnant women with symptoms of post-traumatic stress disorder (PTSD), who have experienced traumatic events such as sexual abuse and traumatic births, are particularly vulnerable to experiencing extreme fear of childbirth complications during labor and traumatic deliveries. In this commentary, we review the literature on this group of women and their specific needs during pregnancy and childbirth. We present a childbirth preparation intervention for pregnant women with PTSD symptoms, “Women Friendly”, designed in Israel and gradually becoming available in the community and Israeli hospitals. This intervention is intended for women with high levels of fear of childbirth who are unmotivated or unable to undergo traditional psychotherapy that focuses on exposure to and processing of past traumatic event(s). It is based on birth-oriented thinking, principles of positive psychology, and trauma-informed care. In addition to the five sessions offered to pregnant women, medical staff are provided with 19 training sessions on the “Women Friendly” approach. Qualitative and quantitative research should examine the effectiveness of this intervention. Should results be encouraging, this intervention could be more widely implemented in Israel and abroad and applied in broader contexts, such as gynecological check-ups and medical examinations, interventions, and surgery.

## 1. Introduction

Most women view pregnancy and childbirth as normative developments that bring with them joy, new life, and meaning, as well as challenges and physical pain. Yet for women who have experienced traumatic events, in particular those related to sexual abuse [1] and previous pregnancies and deliveries [2], the perinatal period is often fraught with tension, fear, and anxiety, and childbirth is an event that is dreaded. In this commentary, we review the literature on this vulnerable group of women and their specific difficulties and needs during pregnancy and childbirth. We then briefly describe an intervention for this group of women that was developed in Israel and is currently implemented there, “Women Friendly”. Finally, we suggest future research to evaluate the effectiveness of this intervention and possible adaptations to broader medical and cultural contexts.

### 1.1. Fear of Childbirth

Since childbirth is a complex, sensitive, and unpredictable process over which the mother has limited control, it is almost universal to be apprehensive about the event to some extent. Fear of childbirth exists on a continuum, with many women experiencing high levels of fear [3]. At its most severe, a specific fear of childbirth has been likened to a phobic response called “tokophobia” (tokos = childbirth in Greek), a term coined by the French psychiatrist Louis Victor Marcé [4], which can seriously affect quality of life [5]. Whether or not tokophobia can be considered a specific phobia, this term is commonly used to describe a severe fear of childbirth [5].

Eriksson & Westman [6] analyzed the open-ended responses of 308 women to a questionnaire about the specific fears they experienced during pregnancy about the childbirth process. The women reported fears about the labor and delivery process (e.g., pain, prolonged labor), the health and life of the baby (e.g., disease or handicap, death), their capabilities and reactions (e.g., not performing “correctly”, losing control of their body), their health and life (e.g., being injured, dying), and professionals’ competence and behavior (receiving inadequate medical care, being treated disrespectfully). Women’s other childbirth-related fears include a lack of support [7], disempowerment and helplessness, a caesarean section, the unknown, and/or the repetition of a traumatic or horrific experience during a past delivery [8]. Fear of childbirth seems to be both a consequence of [9] and a risk factor for [10] symptoms of trauma, and prevention interventions are limited [11].

#### Risk Factors for Fear of Childbirth and Traumatic Birth: History of Abuse and Traumatic Childbirth Experiences

In terms of etiology, many sociodemographic, psychosocial, and personality factors have been linked to an extreme fear of childbirth [12]. Two are worthy of special mention. The first is a history of sexual, emotional, or physical abuse or violence [13,14,15]. Soet et al. [16] found that women who had experienced past sexual trauma were 12 times more likely than women who had not experienced sexual trauma to experience the childbirth event as traumatic. Heimstad et al. [13] found that Norwegian women who reported having been exposed to physical or sexual abuse during childhood scored significantly higher on fear of childbirth than women who had not. Leeners et al. [17] compared the birth experiences of 85 women with a history of child sexual abuse to those of women without such a history. They found that for women who were abused as children, childbirth was a significantly more negative and frightening experience. However, supportive factors during pregnancy and labor, such as birth preparation classes, the presence of a trusted person, and active participation in medical decisions, weakened this effect. Support and empowerment during pregnancy and childhood therefore seem to be of particularly great value to women who were abused as children and should therefore be a central element of childbirth interventions for this population.

A second factor strongly predictive of an extreme fear of childbirth is a previous distressing or traumatic birth experience [2] that may have involved prolonged labor, birth complications, and/or emergency obstetric procedures [18], such as a caesarean section or vacuum extraction [19]. Sexual abuse and traumatic childbirth experiences are also included in the category of sudden and catastrophic events that can lead to the development of post-traumatic stress disorder (PTSD) [20]. Childbirth leads to PTSD in an estimated 3–4% of women, with 15–19% of women in high-risk groups developing postpartum PTSD [21,22]. Pre-existing post-traumatic symptoms may also be exacerbated following pregnancy and childbirth [23,24]. Ertan et al. [25] identified social support as a protective factor for the well-being of mothers with PTSD symptoms following childbirth, underscoring the importance of support in childbirth interventions. McKenzie-McHarg et al. [11] suggested the prevention strategies of early identification of vulnerable women and additional midwifery support to ensure compassionate care during labor.

### 1.2. Mistreatment of Women during Childbirth

Mistreatment of women and violence against them during childbirth are sadly not uncommon [26,27,28]. Perrotte et al. [29] used the seven categories of Bowser and Hill’s [30] seven categories of “disrespect and abuse” or “obstetric violence” (physical abuse, non-consensual care, non-confidential care, non-dignified care, discrimination, inadequate care, and detention in facilities) to review 22 relevant studies on disrespect and abuse of women during childbirth and found this to occur throughout the world. Fear of a repetition of disrespect and abuse in subsequent births causes some women to avoid pregnancy [31], plan unsafe home births, and avoid available obstetric care [32]. Since women often describe experiences of violence in childbirth via metaphors of rape [33], those who have past experience of sexual abuse, especially rape, are particularly prone to re-traumatization when professionals use forceful behaviors during delivery. This tragic state of affairs has led to definitions of and a call for respectful maternity care [34,35].

According to Shabot [33], one of the causes of obstetric violence during childbirth is power imbalances between physicians and patients, in particular when the woman’s choice differs from the decision of the physician. Patients often choose not to challenge the system by reporting obstetric violence since they feel dependent on the medical team for their ongoing and future care [36]. There is, therefore, a great need for change via training and discussion among medical teams, to make them aware of the power they have because of their professional and institutional status, and the need for them to listen carefully to what women want when delivering their babies and to respect their wishes as far as possible. Working conditions in obstetrical wards are often stressful because of low resources, understaffing, and long shifts [37,38], so improving the working conditions of professionals involved in giving birth also stands to contribute to the well-being of women in childbirth.

### 1.3. Vicarious Traumatization and Needs of Medical Staff

Obstetric medical staff members often experience emotionally complex events because of their central and critical role in childbirth. This role can expose them to loss and trauma during pregnancy and childbirth, for which they are ill-prepared and unable to process adequately [39]. Midwives, in particular, are at high risk for symptoms of PTSD for these reasons [40,41]. Secondary traumatic stress resulting from indirect traumatic exposure in a professional context is sadly common in all professionals who help women deliver their babies [42,43,44]. Beck & Gable [45] collected both quantitative and qualitative data from 464 labor and delivery nurses in the US, of whom 35% reported moderate to severe levels of secondary traumatic stress. Content analyses of participants’ descriptions yielded six themes: (a) facing situations that intensify exposure to traumatic births; (b) struggling to maintain a professional front; (c) agonizing over what should have been done; (d) mitigating the aftermath of exposure to traumatic births; (e) secondary traumatic stress symptoms; and (f) considering a change in career. It is therefore of great importance for medical professionals involved in childbirth to receive information, training, and emotional support related to the traumatic aspects of pregnancy and delivery.

De Vries [46] found that midwives generally hold positive attitudes towards women with PTSD but feel that, in general, they lack the knowledge necessary to help them adequately. It is important to offer relevant training to midwives and to all professionals who come into contact with women during pregnancy and childbirth.

### 1.4. The Need for Interventions for Women with Severe Fear of Childbirth

Little is known about how pregnant women with severe fear of childbirth can be best supported and how appropriate support can be best organized within healthcare systems. Respect and joint decision-making seem essential to a positive emotional environment for women giving birth [47,48,49]. As suggested by Reynolds [50] and by Gelaye et al. [51], thorough and discreet screening for past traumatic experiences, particularly sexual trauma and traumatic birth, is important.

Psychoeducation can be effective in reducing the fear of childbirth, preventing re-traumatization, and reducing the use of caesarean sections [19,48,52,53]. Eye Movement Desensitization and Reprocessing (EMDR) is not more effective than care-as-usual in treating fear of childbirth [54]. Hosseini et al. [55] examined the effectiveness of the interventions evaluated in eight studies that used education and two that used hypnosis to reduce fear of childbirth and concluded that educational interventions may be twice as effective as hypnosis. Newham et al. [56] found that 29 women randomized to receive eight weeks of antenatal Hatha yoga reported lower levels of fear of childbirth than 28 women who received treatment as usual, although the effect size was small. Other interventions that may be effective include supportive midwifery [57] and cognitive behavioral approaches, which are effective treatments for anxiety disorders [58]. It is unclear how effective birth plans are in reducing fear of childbirth [59], although Thomson & Downe [60] found that those that help prepare women for multiple realities were perceived as helpful. A Cochrane review synthesizing empirical evidence for non-pharmacological interventions to treat fear of childbirth, including tocophobia, included seven trials with a total of 1357 participants [61]. The authors concluded that the effect of such interventions is uncertain, although there may be a reduction in caesarean section delivery.

There is a growing awareness in Israel of the need to address the emotional as well as medical needs of women in pregnancy and labor, which has given rise to a field coined “Birth-oriented Thinking” (see https://www.bot.co.il/english-bot, accessed on 15 August 2023). This field focuses on the emotional aspects of the pre- and post-partum period and of labor itself. Its overarching aim is to promote mindful communication about birth between women giving birth and the medical team. This is done via education and training programs for professionals involved in childbirth, such as physicians, midwives, psychotherapists, nurses, doulas, and social workers, so as to address the needs of all involved. The “Women Friendly” intervention, described below, is one example of how “Birth-oriented Thinking” programs help women to cope successfully with the perinatal period and labor.

Psychotherapy, including hypnotism, biofeedback, emotional freedom techniques and breathing awareness, group psychotherapy, EMDR, psycho-education intensive therapy, mindfulness, cognitive group therapy, relaxation and directed images, and reality therapy, can, in fact, be helpful for women with fear of childbirth, according to a recent systematic review [53]. However, pregnant women with symptoms of PTSD may not be motivated or emotionally available for psychotherapy that addresses sensitive issues from the past that affect their pregnancy and delivery. In-depth therapy that aims to process past abuse or other traumatic events often lasts longer than nine months and requires inner emotional resources that may not always be available. For these reasons, pregnant women may therefore decide not to engage in therapy during pregnancy to address PTSD symptoms, yet they may experience distress as childbirth approaches, which they would like to be able to manage.

## 2. The “Women Friendly” Intervention

The “Women Friendly” intervention program is an example of birth-related thinking that focuses on women who have experienced traumatic events. It is a childbirth preparation program for pregnant women with symptoms of PTSD, founded in Israel in 2017 by Hila Lev-Ran. This program aims to improve communication between mothers with complex emotional backgrounds and the medical staff during childbirth so as to facilitate as positive an experience as possible all around and prevent the resurgence or exacerbation of traumatic memories in the mothers. The needs of both mothers and the medical staff in the labor wards are therefore targeted. The overall goals are consistent with positive psychology interventions: An emphasis on strengths and sources of support and the empowerment of the pregnant woman to deal with the challenges posed by her traumatic past and the potential triggers of childbirth. The goals of the “Women Friendly” childbirth intervention are also consistent with the principles of trauma-informed care [62,63,64], which aim to integrate an understanding of trauma and trauma-related symptoms into routine practice so as to create a safe and healing environment. The intervention also strives to break the vicious cycles of aggression that can develop during childbirth toward women who have experienced traumatic events. It is offered either as a stand-alone intervention or in combination with medications.

### 2.1. Training for Medical Staff

Since the “Women Friendly” childbirth preparation intervention is delivered by healthcare professionals who accept joint responsibility for the well-being of the women giving birth, they are provided with suitable training.

The “Women Friendly” intervention includes 19 four-hour training sessions over the course of six months, covering theoretical and practical aspects of the intervention for medical staff. This training is offered to midwives, doctors, nurses, technicians, psychologists, social workers, doulas, lactation consultants, and pelvic floor physiotherapists. During the training, professionals are taught skills to be used in checkups, medical procedures, and childbirth, and how to adapt them when needed, based on the personal history presented by each woman.

The skills imparted during the training include how to:

- identify women with post-traumatic symptoms and their needs, including women who have experienced sexual abuse or previous birth trauma.
- engage in conversations in which women relate their past traumatic experiences.
- conduct an initial meeting at the Women Friendly Clinic with women who have had traumatic experiences.
- facilitate the preparation of a personalized birth plan (“Women Friendly” document).
- support women briefly and effectively when they are fearful and anxious during labor.
- speak sensitively to women at all stages of childbirth, e.g., while waiting for an epidural, after giving birth.
- use first aid tools and emotional support in the delivery room.
- self-regulate in emergency situations, such as the need to move from a delivery room to an operating theater, or the need to resuscitate a newborn.
- accompany women during and after complex and traumatic emotional experiences such as stillbirths.

Medical staff in hospitals, who are at the highest risk for vicarious traumatization, are offered a more intensive intervention that takes 130 h, spread over six months. The topics in this intensive course include in vitro fertilization (IVF) units, high-risk pregnancies, labor, and premature deliveries. Specific techniques are imparted for self-regulation in high-arousal emergency situations, for contending with the symptoms of secondary traumatization, for supporting women who have experienced sexual abuse, pregnancy loss, and other acute distress situations, and for mutually supportive communication between staff members.

### 2.2. Childbirth Preparation Intervention for Pregnant Women

Five one-hour, individual sessions over approximately two months are included in the “Women Friendly” intervention for pregnant women with fear of childbirth. They are spread over approximately two months, although this varies according to the stage of pregnancy at the time of the first session and the individual circumstances of each woman. Facilitators are midwives, doctors, nurses, technicians, psychologists, social workers, doulas, lactation consultants, and pelvic floor physiotherapists who successfully completed the training described above.

The first session focuses on psychoeducation about childbirth and teaches cognitive-behavioral techniques that can be used to desensitize the potential PTSD triggers anticipated in the process of childbirth. For example, a woman who previously experienced a traumatic experience during delivery may be encouraged to visit the hospital and, if possible, enter a labor ward to decrease levels of anxiety when exposed to these specific settings.

The second session is devoted to the identification and processing of fears and anxieties associated with the birth process. The group facilitator asks each participant to identify her fears and anxieties and reflect on them, to increase awareness of the challenges that lie ahead.

During the third session, participants delve further into potential specific situations that could lead to stress, distress, and/or re-traumatization during childbirth. The women are encouraged to think of scenarios that might trigger the emergence of post-traumatic symptoms, and troubleshoot and envisage coping strategies.

During the fourth and fifth sessions, participants put in writing, in collaboration with the group facilitator, their “Women Friendly” document. This is a record of personal background and requests that the women would like to convey to the medical team when they arrive at the hospital to give birth; no separate birth plan is prepared. The purpose of the document is to give the women a voice and allow them to communicate their genuine wishes for how they are treated during childbirth, to help them assert themselves rather than be passive and dependent on the hospital staff. Personal information and background about emotional history are presented, although each woman determines the level of self-disclosure with which she feels comfortable. The document describes the potential anxiety- or distress-provoking scenarios identified during the “Women Friendly” sessions and suggests possible strategies for the medical staff to help overcome them and minimize distress. For example, a woman who has identified a vaginal examination by a male doctor or nurse as a potential stressor might request to be examined by women only.

### 2.3. Implementation

To date, 75 female “Women Friendly” facilitators have been trained in Israel. Four hospitals in Israel have established official “Women Friendly” clinics, headed by midwives trained in the “Women Friendly” approach. Women who have participated in a “Women Friendly” childbirth preparation intervention with a professional in the community during pregnancy can prepare and take along a “Women Friendly” document when they give birth in any Israeli hospital. More professionals should be trained in the approach to ensure a positive and supportive response to the “Women Friendly” document across birth settings. The “Women Friendly” approach is gradually expanding beyond labor wards to include other medical contexts. For example, a “Women Friendly” intervention is now available for women who need to undergo invasive medical treatments.

## 3. Suggestions for Research

The feasibility and effectiveness of the “Women Friendly” intervention should be addressed in future research. The experiences of women participating in the intervention could be explored in both qualitative and quantitative research. In a qualitative study, for example, the birth experience of women who participate in the intervention could be explored via face-to-face interviews to investigate its personal benefits and possible drawbacks. The perspectives of partners, other family members, and the medical team would also be of interest.

A quantitative study could compare symptoms of PTSD in women with a history of traumatic experiences who participated in the intervention with women with a history of traumatic experiences who did not. In initial quantitative research evaluating the effectiveness of the “Women Friendly” intervention, it may be advantageous to include only women not taking psychiatric medication, to rule out medication as a possible confounding factor.

Future studies should also compare the symptoms of PTSD in medical staff before and after their participation in the intervention. The health outcomes of the offspring of women with PTSD who participated in the intervention could also be compared to those of the offspring of women with PTSD who did not participate in the intervention. Finally, it should be examined whether “Women Friendly” is appropriate for use across cultures.

Should research provide evidence for the effectiveness of this intervention, the program could be expanded to more hospitals and therapists, encouraged by gynecologists, nurses, and other professionals during pregnancy, and implemented in other countries and cultures. The principles of this childbirth intervention could also be applied in broader contexts, such as gynecological check-ups, medical examinations, interventions, and surgery. It could also be used in additional medical contexts and settings, such as maternity wards following birth, routine gynecological examinations, and other medical examinations.

## Data Availability

Not applicable.
